# Antimicrobial Drug Prophylaxis for Recurrent Ocular Toxoplasmosis

**DOI:** 10.3390/pathogens15040388

**Published:** 2026-04-04

**Authors:** Taraneh Keshavarz, João M. Furtado, Justine R. Smith

**Affiliations:** 1 College of Medicine and Public Health, Flinders Health and Medical Research Institute, Flinders University, Adelaide, SA 5042, Australia; taraneh.keshavarz@flinders.edu.au; 2 Division of Ophthalmology, Ribeirão Preto Medical School, University of São Paulo, Ribeirão Preto 14049-900, SP, Brazil; furtadojm@gmail.com

**Keywords:** ocular toxoplasmosis, prophylaxis, antimicrobial

## Abstract

Ocular toxoplasmosis is a relapsing infectious eye disease that carries an increasing risk of vision loss with each reactivation episode. Antimicrobial drug prophylaxis has been used to reduce the rate of recurrence. This review aims to summarize the current literature regarding expert clinician preferences, as well as the effectiveness and safety of prophylaxis. A literature search was conducted using the PubMed platform of the National Library of Medicine of the National Center for Biotechnology Information and relevant pre-specified search terms. Four professional surveys indicated that approximately three-quarters of experts gave antimicrobial drug prophylaxis for recurrent ocular toxoplasmosis, and that trimethoprim-sulfamethoxazole was the most popular approach. Clinical studies of prophylaxis varied in multiple parameters, including drug, dosing and duration, plus time of follow-up. Considering the four studies with at least 50 participants, the rate of recurrence of ocular toxoplasmosis within 5 years was up to 9.1% of patients taking prophylaxis, and treatment-limiting side effects occurred in up to 7.9% of patients. The available literature demonstrates that antimicrobial drug prophylaxis can reduce the recurrence rate of ocular toxoplasmosis; however, further research on drug dosing and duration of treatment is required to assist decision-making in clinical practice.

## 1. Introduction

Ocular toxoplasmosis is a parasitic eye disease caused by *Toxoplasma gondii* that represents the leading cause of posterior uveitis worldwide [1,2] and imposes a considerable burden on vision-related quality of life [3,4]. A central challenge in managing ocular toxoplasmosis, also referred to as toxoplasmic retinochoroiditis, is that commercially available drugs do not eliminate tissue cysts. After the primary infection, viable dormant bradyzoites persist indefinitely in the retina, with the potential for conversion to highly replicative tachyzoites and reactivation of the disease [5]. Although it is long-established that those with recently active ocular episodes, immunocompromised persons, and older adults are at higher risk of a new episode of active toxoplasmosis [6], many aspects of why and when reactivation occurs remain unknown. As a result, ophthalmologists deal with a disease that is controlled, but not cured, and each recurrence carries the potential for irreversible vision loss either due to retinal scars or other complications [7].

Over the last 2 decades, the use of antimicrobial drug prophylaxis has become more common as a strategy to lower the rate of recurrence of ocular toxoplasmosis [8]. This approach aims to maintain a low, continuous systemic concentration of an anti-parasitic drug that can act early to limit and/or reverse bradyzoite-to-tachyzoite conversion before clinical symptoms and signs appear [9]. Despite its growing popularity, there are knowledge gaps across the studies on this subject, including limited comparative data on drug regimens and duration of treatment, and practical concerns related to drug toxicity.

In this review, we have synthesized the published peer-reviewed literature supporting antimicrobial drug prophylaxis for recurrent ocular toxoplasmosis and examined its limitations, summarizing the existing available data on drug selection, duration of use, and reported outcomes. Articles were identified through a systematic search conducted on the PubMed platform of the National Library of Medicine of the National Center for Biotechnology Information using relevant keywords. Against this background, we also have discussed gaps in the literature and priorities for future research.

## 2. Literature Search

A literature search was conducted on 7 November 2024, and updated on 9 December 2025, for articles that addressed the question: How effective and safe is antimicrobial drug prophylaxis for recurrent ocular toxoplasmosis? Antimicrobial drug prophylaxis was defined as the use of drugs to reduce the risk of recurrence of toxoplasmic retinochoroiditis in the absence of active inflammation. The question was focused specifically on secondary prophylaxis, not prevention of primary infections.

The PubMed platform of the National Library of Medicine of the National Center for Biotechnology Information was searched to identify articles that were relevant to the stated question, applying the search terms: “*ocular toxoplasmosis prevention*,” “*ocular toxoplasmosis prophylaxis*,” “*toxoplasmic retinitis prevention*,” “*toxoplasmic retinitis prophylaxis*,” “*toxoplasmic retinochoroiditis prevention*,” “*toxoplasmic retinochoroiditis prophylaxis*,” “*toxoplasmic chorioretinitis prevention*,” and “*toxoplasmic chorioretinitis prophylaxis*”. No filters or restrictions were applied. Articles were identified according to the following criteria: inclusion: articles describing clinical studies of, or expert preferences on, the use of antimicrobial prophylaxis for recurrent ocular toxoplasmosis; exclusion: articles that did not report on the use of antimicrobial drug prophylaxis for recurrent ocular toxoplasmosis, including, but not limited to, articles reporting in vitro studies, animal studies, and studies of congenital toxoplasmosis, perinatal treatment, and non-pharmacological treatments. Articles in languages other than English were translated with Google Translate.

Titles and abstracts of articles identified by the literature search were reviewed independently by two authors (T.K. and J.R.S.). A manual search of reference lists of relevant review articles was also undertaken by these two authors. The full texts of the selected articles were examined separately by two authors (T.K. and J.M.F. or J.R.S.), and those articles identified as relevant to the stated question were identified. Pertinent information was manually extracted from the articles, including: study location, study type, participant or patient population, antimicrobial drug choice, dosing schedule and duration, effectiveness of prophylaxis, and drug adverse events (extracted by T.K., verified by J.M.F. and/or J.R.S.). Differences in opinion on selection of articles or extraction of information were resolved by discussion between the authors. The level of the medical evidence presented in the articles was rated using the American Academy of Ophthalmology grading guidance that has been used for Ophthalmic Technology Assessments including drug therapy of ocular toxoplasmosis [10]; if there was more than one article per study, these were considered together in determining the rating.

## 3. Findings

### 3.1. Overview of the Published Literature

**Figure 1:**
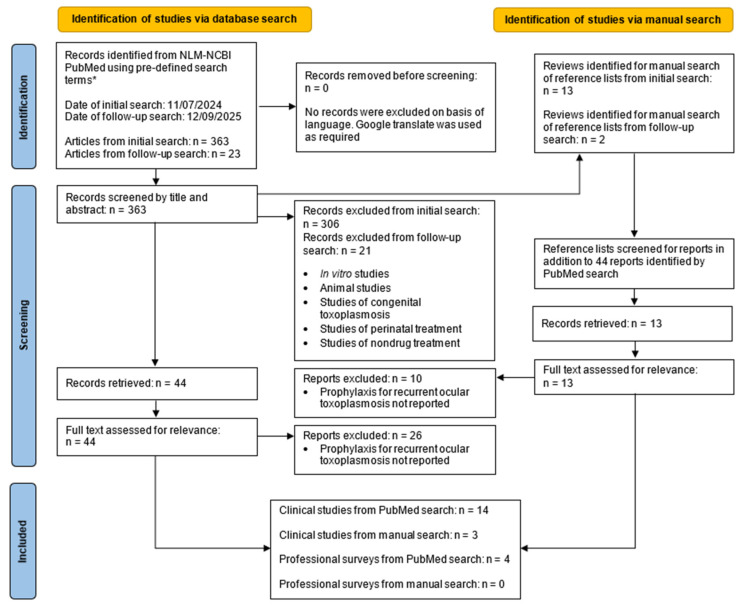
PRISMA flow diagram of the literature search strategy and results on the topic of antimicrobial drug prophylaxis for recurrent ocular toxoplasmosis. * Search terms are listed in Section 2.

The initial PubMed-based search identified 363 articles, and the update search yielded a further 23, giving a total of 386 articles. Overall, 44 articles were identified as potentially relevant to the question: How effective and safe is antimicrobial drug prophylaxis for recurrent ocular toxoplasmosis? In addition, 15 review articles were identified for a manual search of the reference lists, and a further 13 articles were identified as potentially relevant through that search. The full texts of these 57 articles were analyzed, and 21 were determined to contain information that was relevant to the research question. Of these 21 articles, 19 were in English, one was in French, and one was in German. In total, 17 articles addressed effectiveness and/or safety of antimicrobial drug prophylaxis against recurrent ocular toxoplasmosis, and four articles presented expert opinions about prophylaxis. The literature search strategy and its results are presented in Figure 1.

### 3.2. Surveys of Practice Patterns

The opinions of clinician experts on the use of antimicrobial drug prophylaxis for recurrent ocular toxoplasmosis are summarized in Table 1. Four articles presented the results of four professional surveys that were addressed to ophthalmologists involved in the management of ocular toxoplasmosis, and that included questions on the circumstances in which these clinicians prescribed prophylaxis, plus their prescription preferences. Three of the four articles were published within the last 5 years, reflecting current practice patterns. One article was from Brazil, two were from Western Europe, and one drew on an international clinician base across 48 countries. The number of clinician respondents ranged from 19 to 192 across the surveys, with a median of 54.

Overall, the majority of surveyed clinicians (79.63%) were willing to prescribe antimicrobial drug prophylaxis to patients with ocular toxoplasmosis. The most common indication was frequent recurrences, with 81.3% of clinicians prescribing prophylactic drugs in this setting. The definition of frequent recurrence was not consistently provided, and it varied between the studies. Schaeffer et al. [12] defined it as two or more episodes in 1 year. In contrast, Taghavi-Eraghi et al. [13] defined it as recurrence within 2 years of acute ocular toxoplasmosis. Similarly, Yogeswaran et al. [8] defined it as a recurrence within the past 2 years. Sight-threatening toxoplasmic retinal lesions were another common indication for prophylaxis. Schaeffer et al. [12] reported that 43.8% of clinicians provided prophylaxis for sight-threatening lesions from the first clinical encounter. The timing of prescriptions was not explored in the other surveys, in which a mean 74.3% of the clinicians provided prophylaxis for sight-threatening lesions. Yogeswaran et al. [8] further identified that 59.3% of clinicians gave prophylaxis in patients who were blind in their other eye. Patient immune status also determined clinician preference for prophylaxis: on average, 77.9% of clinicians prescribed prophylaxis if the patient was immunocompromised.

The most popular drug of choice for preventing recurrences of ocular toxoplasmosis was trimethoprim-sulfamethoxazole, with 89.8% of clinicians using it as their first preference. Dosing regimens were not reported. Three surveys did not specify the duration of treatment, but Yogeswaran et al. [8] found that 84.1% of clinicians preferred to continue prophylaxis for more than 6 months.

### 3.3. Clinical Reports

Results of 17 articles relevant to the effectiveness and/or safety of prophylactic antimicrobial drug treatment were collated, as summarized in Table 2. The articles showcased research from four continents, with majority of the articles being from Brazil, followed by France, Poland, and the United States. Other locations included Australia, Canada, Germany, and the United Kingdom. The oldest included article was a case series by Linton et al. [14] from 1969, and the most recent was a case report by Amato et al. [15] from 2024. Five articles described two randomized controlled trials (rated as level I evidence), both conducted in Brazil, by Fernandes Felix et al. [16,17,18] and Silveira et al. [9,19]. Two articles reported a cohort study [20,21] (rated as level II evidence), and there were six case series [14,22,23,24,25,26] and four case reports [15,27,28,29] (rated as level III evidence). A pooled total of 545 patients were studied. The number per study ranged from 1 to 314 patients, with a mean of 42 and a median of 3. Due to reported observations being made in the same study populations, the articles by Fernandes Felix et al. [16,17,18], Silveira et al. [9,19], and Borkowski et al. [20,21] were interpreted as a single study each. Regarding patient immune status, seven studies included only immunocompetent individuals [15,18,19,21,23,24,26], three included immunocompromised individuals [27,28,29], and the remainder did not specify [14,25].

### 3.4. Drug Prophylaxis

All studies reported an antimicrobial drug of choice for prophylaxis against recurrent ocular toxoplasmosis. The following drug combinations were examined across the 13 studies: trimethoprim-sulfamethoxazole (eight studies) [18,19,22,23,24,27,28,29], pyrimethamine (two studies) [14,15], pyrimethamine and sulfadoxine (one study) [21], pyrimethamine with folinic acid (one study) [26], and doxycycline (one study) [25]. Notably, the popularity of trimethoprim-sulfamethoxazole in the professional surveys [8,11,12,13] was reflected in the drug choice in these studies. No study provided objective data on adherence to the drug treatment.

In seven of the eight studies that reported on trimethoprim-sulfamethoxazole prophylaxis [18,19,22,23,24,27,28], the dose was specified as 160 mg–800 mg. Silveira et al. [9,19] were the only team that provided a separate pediatric dose: 0.375 mL/kg of trimethoprim (40 mg/5 mL)-sulfamethoxazole (200 mg/5 mL). There was no consensus amongst the studies on the dosing frequency, which varied as every 3 days [9,19], every 2 days [16,17,18], 2 consecutive days per week [27], three times per week [22], once per day [24,28], and twice per day [23]. In three studies focused on pyrimethamine prophylaxis, the drug was dosed at 25 mg, either alone [14,15] or with 15 mg of folinic acid [26]. Additionally, in the two articles describing their cohort study, Borkowski et al. [20,21] alternatively reported doses of 25 mg and 50 mg of pyrimethamine, combined with 500 mg and 1000 mg of sulfadoxine, respectively. No pediatric dose for pyrimethamine was provided. Again, dosing frequencies were quite variable, including once a week [14], twice a week [20,21], thrice a week [26], and once every other day [15]. Uniquely, Saad et al. [25] reported on the use of doxycycline at 100 mg daily in a two-patient series, although this was prescribed with the initial intention of prophylaxis against malaria. The duration of therapy varied amongst the nine studies in which this was specified [15,18,19,21,22,24,25,26,28]. Prophylaxis was prescribed for intervals as short as 2 weeks [22] and as long as 4 years [28], with a mean duration of 12 months. This wide variation in dosing frequency and duration of treatment across the studies made it difficult to synthesize these data and generate conclusions about the effectiveness and safety of antimicrobial drug prophylaxis.

### 3.5. Reported Effectiveness

The main outcome measure for all but two articles was the recurrence of ocular toxoplasmosis. Borkowski et al. measured ‘recurrence-free rate’ in their 2016 publication [20], and focused on the adverse events of pyrimethamine and sulfadoxine prophylaxis in their 2018 publication [21]. Their efficacy measure was converted to a recurrence rate to permit synthesis in this review. In addition to the different treatment approaches across the studies, the duration of surveillance on and after antimicrobial drug prophylaxis was highly variable, ranging from 1 year [15] to 10 years [19], or not specified. This posed a substantial challenge for summarizing long-term effectiveness. Of the 13 studies focusing on recurrence, two did not quantify the recurrence rate. Matet et al. [24] reported no reduction in recurrence rate between trimethoprim-sulfamethoxazole-prophylaxed and non-prophylaxed groups, but numerical values were not provided. Linton et al. [14] reported that at least eight individuals presented with recurrences after discontinuation of prophylaxis, but the denominator was not given. Six studies, all case reports or small case series, reported no recurrences within the surveillance period [15,22,23,27,28,29]. However, Cavattoni et al. [27] documented detectable *T. gondii* immunoglobulin M in the absence of clinical symptoms of recurrence in their case report. In their randomized placebo-controlled trial, Fernandes Felix et al. observed no recurrences in the therapy group versus 26.1% in the placebo group up to 5 years after the start of drug treatment [17]. At 6 years, one participant had a recurrence, equating to 1.4%, which was considerably less than the 27.5% recurrence rate for the placebo group [18]. Three additional large studies gave recurrence rates of less than 10% [9,20,26]. While initially reducing the recurrence rate from 23.8% to 6.6% by 20 months of antimicrobial drug treatment [9], in the 10-year follow-up report of their randomized observation-controlled trial, Silveira et al. [19] found similar recurrence rates for therapy and control groups: 37.3% and 38.6%, respectively. Saad et al. reported that two patients treated with doxycycline developed recurrent ocular toxoplasmosis [25]. Notably, this was the only study in which prophylaxis against ocular toxoplasmosis was not the primary intention of treatment.

To estimate a rate of recurrence of ocular toxoplasmosis in patients taking antimicrobial drug prophylaxis, the four studies with at least 50 participants and specified durations of follow-up were considered [9,16,17,18,19,20,26]. The following rates of recurrence were reported: 0% in year 1 in one randomized controlled trial [16,17]; up to 6.6% in year 2 in two randomized controlled trials and one large case series [9,17,26]; up to 9.1% in year 3 in one randomized controlled trials and one cohort study [17,20]; 0.0% in year 4 in one randomized controlled trial [18]; 0.0% in year 5 in one randomized controlled trial [18]; 1.4% in year 6 in one randomized controlled trial [18]; and 37.3% in year 10 in one randomized controlled trial [19]. The two randomized controlled trials tested the effectiveness of trimethoprim-sulfamethoxazole [18,19], yielding recurrence rates of 6.6% within 2 years (versus 23.8% in untreated controls) [9] and 1.4% within 6 years (versus 27.5% in placebo-treated controls) [18]. The large case series and the cohort study examined the effectiveness of pyrimethamine [20,26], giving recurrence rates of 4.8% within 1.5 years [26] and 9.1% within 3 years [20], respectively.

### 3.6. Reported Adverse Events

Adverse events caused by the antimicrobial drug prophylaxis were described in nine of the 17 articles [9,14,15,16,17,20,21,26,29], reflecting seven of the 13 studies. Across these studies, patients described in one case report and one small case series had no reported adverse drug events [14,15], patients in two randomized controlled trial and one cohort study had events in less than 10% [9,17,21], patients in one large case series had events in 20.6% [26], and the patient in another case report suffered complications [29]. All four studies of pyrimethamine considered adverse events [14,15,21,26], whereas only three of eight studies using trimethoprim-sulfamethoxazole considered them [9,17,29]. In two of four studies using pyrimethamine, adverse events were observed [21,26]. In all three studies using trimethoprim-sulfamethoxazole, adverse events were recorded [9,17,29]. Premature discontinuation as a result of drug complications was addressed in seven articles [9,16,17,20,21,26,29] or five studies. Fernandes Felix et al. reported no premature terminations, whilst also observing low rates of adverse events in their randomized controlled trial [17]. In the randomized controlled trial by Silveira et al., as well as one cohort study and one large case series, there was up to 7.9% treatment termination due to adverse events [9,21,26], and one patient described in a case report also ceased treatment for drug complications [29]. Considering only the four studies with at least 50 participants, the mean reported rate of adverse drug events was 8.7% of treated patients, and treatment-limiting side effects were reported in up to 7.9% [9,17,21,26].

The adverse events associated with antimicrobial drug prophylaxis may be divided by body system. The most commonly affected system was the gastrointestinal tract, with symptoms that included vomiting (7.9%) [26], mild epigastric burning (2.8%) [17], gastric upset (≥1.6%) [21], and abdominal pain (≥0.3%) [21]. Hepatoxicity resulted in elevated liver function tests (3.2%) [26] and elevated alanine aminotransferase (≥2.7%) [21]. Dermatological involvements comprised cutaneous erythema (6.6%) [9] and hypersensitivity reactions (≥1.1%) [21]. Hematological disturbances were also reported, including myelosuppression (1 case report) [29], mild hematological changes (1.6%) [26], and thrombocytopenia (≥0.3%) [21]. One study recorded elevated serum creatinine (3.2%) [26]. Adverse events that led to discontinuation of the drugs were: changes in blood chemistry or cell parameters, abdominal pain, skin reactions, and myelosuppression [9,21,26,29]. Both trimethoprim-sulfamethoxazole and pyrimethamine regimens showed potential to cause adverse events affecting gastrointestinal, cutaneous, and hematological systems. Trimethoprim-sulfamethoxazole was associated with epigastric discomfort, cutaneous erythema, and myelosuppression [9,17,29]. Pyrimethamine was associated with hematological changes such as thrombocytopenia, gastrointestinal symptoms including vomiting and abdominal pain, deranged liver and renal function markers, as well as cutaneous manifestations [21,26].

## 4. Discussion

Our synthesis of the current literature indicates that antimicrobial drug prophylaxis for recurrent ocular toxoplasmosis is widely used despite there being relatively few published studies that address this subject. Across professional surveys, most clinicians favored trimethoprim-sulfamethoxazole and tended to prescribe it for several months, usually to patients considered to be at higher risk for recurrence [8,11,12,13]. The two randomized controlled trials, both conducted in Brazil, reported substantial reductions in recurrence during the first years of follow-up, suggesting that sustained treatment with antimicrobial drugs may reduce the chances of ocular reactivations [9,16,17,18,19].

There are gaps in the published literature. None of the studies cited in this review objectively measured compliance of participants with the antimicrobial drug therapy, and this may have been overestimated. In the four largest studies, extended courses of treatment were prescribed, ranging from 6 to 20 months [9,16,20,26]. With patients having no symptoms to reinforce motivation, and the potential for adverse events, there is particularly high likelihood of non-compliance with antimicrobial drug prophylaxis for recurrent ocular toxoplasmosis. Poor adherence can bias recurrence estimates and may partially explain divergent results across studies. Some clinically relevant toxicities—cutaneous reactions, bone marrow suppression, and renal dysfunction—were observed [9,21,26], but the frequency of these events in broader populations remains uncertain. Data in children are even less reliable, as dosing regimens are frequently extrapolated from adults, and tolerability issues such as palatability and the burden of monitoring are poorly documented.

Important practical questions remain unaddressed. The optimal duration of antimicrobial drug prophylaxis is unknown, and the tendency to extend treatment beyond 1 year is driven more by clinical habit than evidence. The global reliance on a small number of trials—especially the ground-breaking 2002 clinical trial by Silveira et al. [9]—to guide drug choice and posology reflects a research gap that has persisted for 2 decades. Alternatives to trimethoprim-sulfamethoxazole are few, and the evidence supporting them is weak [26]. There are no validated strategies for patients with chronically poor adherence, no long-acting formulations, and no comparative data on generic versus reference drug effectiveness. Cost considerations are also unevaluated. Even though the drugs themselves may be inexpensive in many countries, the cumulative indirect costs of repeated laboratory monitoring, clinic visits, and management of adverse events affect feasibility, particularly in resource-constrained settings. The contribution of long-term antibiotic exposure to antimicrobial resistance also has not been quantified.

Given these uncertainties, broad prophylaxis against recurrent toxoplasmic retinochoroiditis is difficult to justify. On the other hand, a targeted approach for patients with clearly elevated risk—such as recent recurrent episodes, sight-threatening lesion locations, monocular status, older patients, and individuals willing to take treatment long-term, including those whose anxiety about recurrence meaningfully affects decision-making—may be reasonable.

The limitations of the current literature on antimicrobial drug prophylaxis for ocular toxoplasmosis are substantial: a small number of randomized controlled trials; geographic concentration of high-quality evidence in one region (Latin America); scarce data from pediatric and immunocompromised patients; and no quantification of compliance. Even so, the existing studies suggest that prophylaxis can reduce recurrence rates in selected patients with tolerable toxicity in most settings. To clarify the role of prophylaxis, future work should include controlled trials comparing shorter and longer durations, pragmatic studies incorporating adherence monitoring, evaluations of simplified regimens or alternative drugs, and development of delivery strategies that do not depend heavily on patient compliance. A better understanding of host and parasite factors influencing recurrence risk also would allow for a more rational identification of patients who may benefit from prophylaxis. Prospective safety reporting, especially in children, remains needed.

## 5. Conclusions

There is limited high-level evidence around the use of antimicrobial drugs to prevent recurrence of ocular toxoplasmosis. Moreover, a fair proportion of the clinical studies on this subject is from one geographic region, making it challenging to accurately assess the role of prophylaxis in clinical practice globally. The published literature indicates antimicrobial prophylaxis can provide a reduction in recurrence rates of ocular toxoplasmosis. Whilst there are some treatment-limiting adverse events, the rate of serious side effects appears to be relatively low compared to the treatment of active disease [30]. Despite this, the potential benefits and possible harms should be considered before initiating antimicrobial drug prophylaxis for recurrent ocular toxoplasmosis.

## Figures and Tables

**Table 1:** Expert opinions on antimicrobial drug prophylaxis for recurrent ocular toxoplasmosis as collected by professional surveys.

First Author, Year [Ref]	Survey Location	Number of Respondents	Prophylaxis Given % (Number/Total)	Indication % (Number/Total)				Prophylaxis	
				Frequent Recurrences	Sight- Threatening Lesion	Patient Blind in Other Eye	Immunocompromised Patient	Drug of Choice % (Number/Total)	Duration
Morais, 2018 [11]	Brazil	54	83.3 (45/54)	83.3 (45/54)	61.1 (33/54)	NR	NR	Trimethoprim/sulfamethoxazole 83.3 (45/54)	NR
Schaeffer, 2022 [12]	France	19	84.2 (16/19)	84.2 (16/19)	43.8 (7/16)	NR	75.0 (12/16)	Trimethoprim/sulfamethoxazole 93.8 (15/16)	NR
Taghavi-Eraghi, 2023 [13]	Germany	53	75.5 (40/53)	75.5 (40/53)	75.5 (40/53)	NR	80.0 (40/50)	Trimethoprim/sulfamethoxazole NR	NR
Yogeswaran, 2023 [8]	48 countries	192	75.5 (145/192)	82.1 (119/145)	86.2 (125/145)	59.3 (86/145)	78.6 (114/145)	Trimethoprim/sulfamethoxazole 92.4 (134/145)	>6 m

Abbreviations: m = months, NR = not reported, Ref = reference.

**Table 2:** Effectiveness and safety of antimicrobial drug prophylaxis for recurrent ocular toxoplasmosis.

First Author, Year [Ref]	Location	Study Type (Level of Evidence *)	Participants		Intervention		Effectiveness (% If Stated)	Adverse Events (% If Stated)		
			Number	Immune Status	Drug and Dosing	Duration		Reported	Premature Discontinuation of Drug	Type
Amato, 2024 [15]	Brazil	Case report (level III)	1	Immunocompetent	Pyrimethamine 25 mg PO every 2 d	1 y	No recurrence during 1-y follow-up	No hematological changes	NR	NR
Borkowski, 2016 [20] **	Poland	Cohort study (level II)	303	Immunocompetent	Pyrimethamine 25 mg PO plus sulfadoxine 500 mg PO 2x/w	6 m	90.9% recurrence-free at 3 y	2.3%	2.3%	NR
Borkowski, 2018 [21] **			314		Pyrimethamine 50 mg PO plus sulfadoxine 1000 mg PO 2x/w		NA	4.9%	2.7%	Elevated ALT (≥2.7%) Hypersensitivity skin reaction (≥1.1%) Abdominal pain (≥0.3%) Thrombocytopenia (≥0.3%)
Cavattoni, 2010 [27]	Germany	Case report (level III)	1	Immunocompromised	Trimethoprim-sulfamethoxazole 160 mg–800 mg PO 2x/w	NR	No recurrence but anti-*T. gondii* IgM detected	NR	NR	NR
Fernandes Felix, 2014 [16]	Brazil	RCT (level I)	Prophylaxis: 47 Placebo: 48	Immunocompetent	Trimethoprim-sulfamethoxazole 160 mg–800 mg PO every 2 d	12 m ***	Recurrence at 1 y: Prophylaxis: 0% Placebo: 12.8%	No treatment limiting toxicity	0%	NR
Fernandes Felix, 2016 [17]			Prophylaxis: 72 Placebo: 69			311 d	Recurrence at 1, 2, 3 y: Prophylaxis: 0%, 0%, 0% Placebo: 13.0%, 17.4%, 20.3%	2.8%	0%	Mild epigastric burning (2.8%)
Fernandes Felix, 2020 [18]							Recurrence at 4, 5, 6 y: Prophylaxis: 0%, 0%, 1.4% Placebo: 23.2%, 26.1%, 27.5%	NA	NA	NA
Hébert, 2022 [22]	Canada	Case series (level III)	3	NR	Trimethoprim-sulfamethoxazole 800 mg–160 mg PO 3x/w	2 w	No recurrence after COVID vaccination	NR	NR	NR
Kopec, 2003 [23]	United States	Case series (level III)	2	Immunocompetent	Trimethoprim-sulfamethoxazole 160 mg–800 mg PO 2x/d	NR	No recurrence during 18 m follow-up	NR	NR	NR
Linton, 1969 [14]	Australia	Case series (level III)	15	NR	Pyrimethamine 25 mg PO 1x/w	NR	Recurrence in at least 8 patients after drug ceased	None	NR	NR
Matet, 2019 [24]	France	Case series (level III)	Prophylaxis: 9 No prophylaxis: 35	Immunocompetent	Trimethoprim-sulfamethoxazole 800 mg–160 mg PO every d	3 m	Same recurrence rate for prophylaxis and no prophylaxis groups	NR	NR	NR
McDermott, 2019 [28]	United States	Case report (level III)	1	Immunocompromised	Trimethoprim-sulfamethoxazole 800 mg–160 mg PO every d	4 y	No recurrence during 4-y follow-up	NR	NR	NR
Saad, 2018 [25]	France	Case series (level III)	2	NR	Doxycycline 100 mg PO every d ****	1 m	Recurrence in 2 patients	NR	NR	NR
Silveira, 2002 [9]	Brazil	RCT (level I)	Prophylaxis: 61 No treatment: 63	Immunocompetent	Adult: Trimethoprim-sulfamethoxazole 160 mg–800 mg PO every 3 d Child: Trimethoprim (40 mg/5 mL)/-sulfamethoxazole (200 mg/5 mL) 0.375 mL/kg PO every 3 d	20 m	Recurrence at 20 m: Prophylaxis: 6.6% No treatment: 23.8%	6.6%	6.6%	Cutaneous erythema (6.6%)
Silveira, 2015 [19]			Prophylaxis: 59 No treatment: 57				Recurrence at 10 y: Prophylaxis: 37.3% No treatment: 38.6%	NR	NR	NR
Webb, 2016 [29]	United Kingdom	Case report (level III)	1	Immunocompromised	Trimethoprim-sulfamethoxazole (dosing NR)	NR	No recurrence during 2-y follow-up	100.0%	100.0%	Myelosuppression
Zamora, 2024 [26]	Brazil	Case series (level III)	63	Immunocompetent	Pyrimethamine 25 mg plus folinic acid 15 mg PO 3x/w	12 m	4.8% recurrence during 18 m follow-up	20.6%	7.9%	Vomiting (7.9%) Gastric upset (1.6%) Elevated LFTs (3.2%) Elevated creatinine (3.2%) Mild hematological changes (1.6%)

Abbreviations: d = days, IgM = immunoglobulin M, LFTs = liver function tests, m = month(s), NA = not applicable, NR = not reported, PO = per oral, RCT = randomized controlled trial, Ref = reference, w = week(s), y = year(s). * Level of evidence was rated using the American Academy of Ophthalmology grading guidance [10]. ** Percentages reported for this study were generated from 352 records of 303 patients [20] and 366 records of 314 patients [21]. *** Two patients (one in the prophylaxis group and one in the placebo group) were lost to follow-up in the course of the study. **** Indication for treatment was prophylaxis against malaria.

## Data Availability

No new data were created for this article.
